# Two novel *TMEM67* variations in a Chinese family with recurrent pregnancy loss: a case report

**DOI:** 10.1186/s12920-024-01902-x

**Published:** 2024-06-06

**Authors:** Jialun Pang, Fanjuan Kong, Wanglan Tang, Hui Xi, Na Ma, Xiaoqi Sheng, Ying Peng, Zhiyu Liu

**Affiliations:** 1 Department of Medical Genetics, Maternal and Child Health Hospital of Hunan Province, 58 Xiangchun Road, Changsha, 410078 Hunan China; 2 Medical Record Management Department, Maternal and Child Health Hospital of Hunan Province, Changsha, Hunan China; 3 NHC Key Laboratory of Birth Defect for Research and Prevention, Maternal and Child Health Hospital of Hunan Province, Changsha, Hunan China; 4 Appropriate Technology Extension Training Centre, Maternal and Child Health Hospital of Hunan Province, Changsha, Hunan China

**Keywords:** *TMEM67*, Exome sequencing, Recurrent pregnancy loss, Meckel syndrome

## Abstract

**Background:**

Recurrent pregnancy loss (RPL) is a common pregnancy complication that brings great pain to pregnant women and their families. Genetic factors are an important cause reason of RPL. However, clinical research on monogenic diseases with recurrent miscarriage is insufficient.

**Case presentation:**

Here we reported a Chinese family with RPL and genetic analysis of the abortion and parents. A paternally inherited heterozygous missense variant c.1415T > G (p.V472G) and a maternally inherited heterozygous nonsense variant c.2314del (p.M772*) in *TMEM67* gene were identified by trio-exome sequencing. c.2314del (p.M772*) generated a premature stop codon and truncated protein, was classified as “pathogenic”. c.1415T > G (p.V472G) located in extra-cellular region, was classified as “likely pathogenic”. Biallelic variants in *TMEM67* gene cause lethal Meckel syndrome 3, consistent with the proband’s prenatal phenotype.

**Conclusion:**

The current study of the Chinese family expands the pathogenic variant spectrum of *TMEM67* and emphasizes the necessity of exome sequencing in RPL condition.

## Background

Recurrent pregnancy loss (RPL) is a devastating reproductive health burden, affects about 3–5% of couples trying to conceive globally [1]. The etiology of RPL is complex, involving genetic abnormalities, metabolic disorders, autoimmune diseases, and endometrial dysfunction [2, 3]. The causes reason of RPL are still unknown in more than 50% of these couples. With the development of detection technology, genetic factors were found may play an essential role in unexplained RPL, such as chromosome abnormalities, copy number variants, single-gene changes and others [2]. Previous researches reveal that the incidence of chromosome abnormalities in sporadic pregnancy loss was significantly higher than RPL [4]. The percent of parental karyotype abnormalities in RPL is 2.25% [5]. The research on copy number variants and single-gene changes in abortions offers a diagnosis for a significant proportion of couples experiencing recurrent pregnancy loss (RPL) [3].

*TMEM67* gene (OMIM #609,884) encode the transmembrane protein meckelin, is a cilia gene associated with a spectrum of ciliopathy phenotypes. Meckel syndrome3 (MKS3, OMIM #607,361) is the most severe ciliopathy leading to lethal in utero or in the perinatal period. MKS3 is an autosomal recessive disorder characterized by bilateral renal cystic dysplasia, occipital encephalocele, hepatic ductal proliferation, fibrosis and cysts, and polydactyly [6].

In this report, trio copy number variation sequencing (CNV-seq) and trio-whole exome sequencing (WES) were performed in the abortion and parents, aim to identify the genetic cause reason of the Chinese family with RPL.

## Case presentation

### Clinical course

**Fig. 1:**
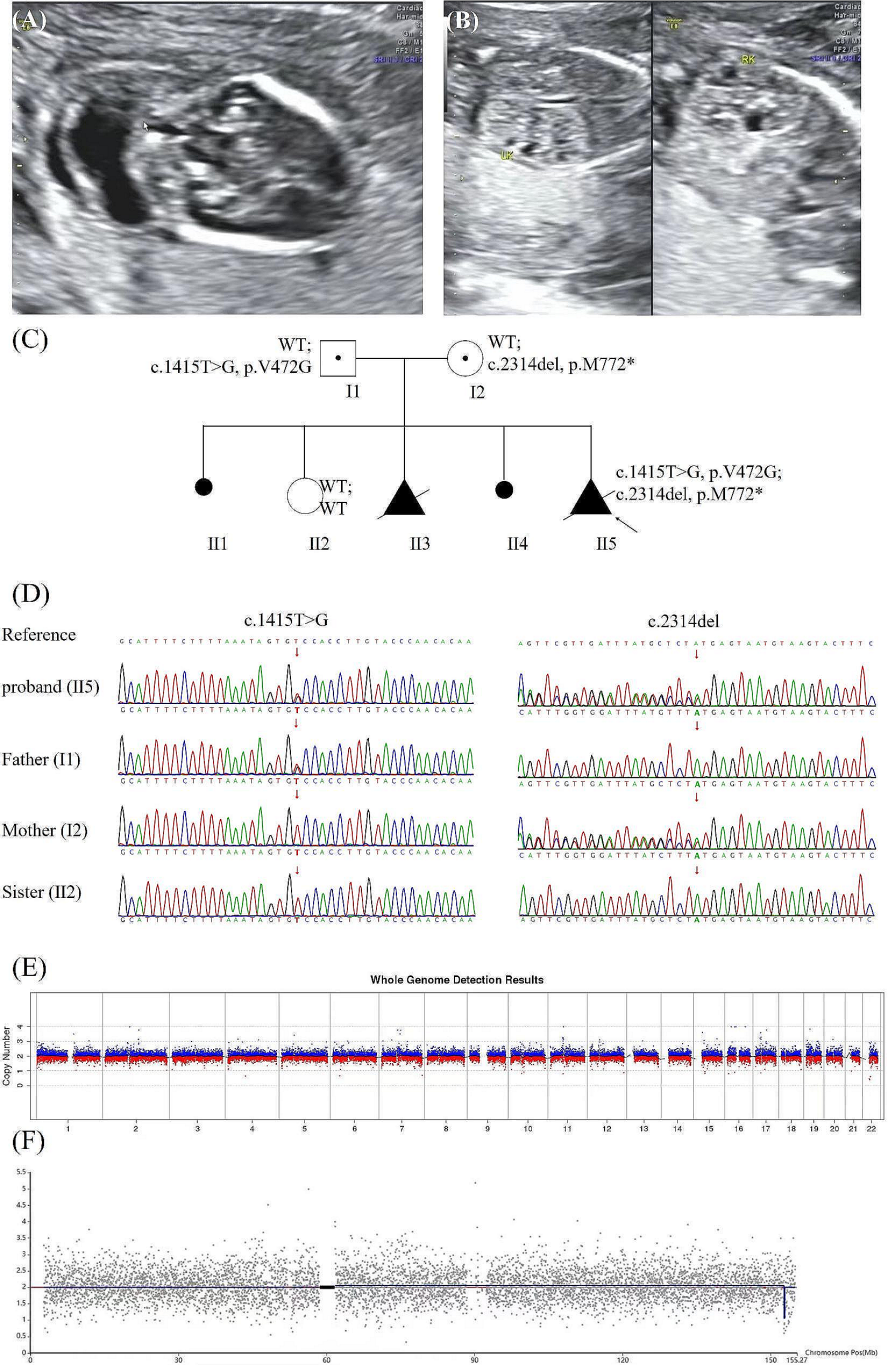
(**A**) Prenatal ultrasound of the proband with polycystic kidney dysplasia at 16 weeks. (**B**) Prenatal ultrasound of the proband with encephalocele at 16 weeks. (**C**) The pedigree of the family: the outcomes of the first and fourth pregnancies were spontaneous abortion, the second pregnancy gives birth to a healthy girl and the third and fifth pregnancies lead to multiple malformations (polycystic kidney dysplasia, oligohydramnios and encephalocele). (**D**) Sanger sequence chromatogram of *TMEM67* gene. Sanger sequencing showed that c.1415T > G (p.V472G) was heterozygous in the proband (II5) and father (I1), and c.2314del (p.M772*) was heterozygous in the proband (II5) and mother (I2). c.1415T > G (p.V472G) was not detected in mother and c.2314del (p.M772*) was not detected in father. Both variants were not detected in the sister (II2). (**E**) CNV-seq results of autosomal chromosomes in the proband. (**F**) CNV-seq results of X chromosome in the proband

A 31-year-old Chinese pregnant woman, G5P1, was admitted to our hospital for recurrent pregnancy loss. There were no relative between the woman and her husband. She experienced twice unexplained spontaneous abortions (II1 and II4) and twice stillbirths (II3 and II5) due to oligohydramnios. Prenatal ultrasound in 16 weeks of II5 showed polycystic kidney dysplasia, encephalocele and oligohydramnios (Fig. 1A, B, C). This was her fifth pregnancy, and she had taken drugs to treat cold under the guidance of a doctor during pregnancy. The clinical symptoms of II3 and II5 were similar, polycystic kidney dysplasia and severe oligohydramnios. She only gave birth to one healthy daughter (II2). The samples from the previous three abnormal pregnancies were not retained. We performed genetic testing on the abortion of the fifth pregnancy and parental samples.

## Genetics analysis

### CNV-seq

Genomic DNA was extracted from the skeletal muscle of the abortion and peripheral blood of the parents using Qiagen DNA Blood Midi/Mini kit (Qiagen GmbH, Hilden, Germany) following the manufacturer’s protocol. Quality of genomic DNA was evaluated by agarose gel analysis and NanoDrop2000. 10ng genomic DNA was treated with NEB Next dsDNA Fragmentase (New England Biolabs, Ipswich, MA, USA) and inputted into the experimental system KR2000 (Berry Genomics, Beijing, China) to generate PCR-free-frag library for sequencing. The sequencing of the libraries was carried out on the NextSeq CN500 platform (Berry Genomics, China) with a run time of 6.5 h. Raw reads were edited to remove artificial adaptor sequences and mapped to the GRCh37 reference genome, which was conducted by the Burrows-Wheeler Alignment (BWA) tool [7]. Reads were processed and copy number variants (CNVs) were evaluated by an in-house pipeline using read counts based on a smoothness model (Berry Genomics, Beijing, China) [8]. Identified CNVs were interrogated the databases including ClinGen, Decipher, Database of Genomic Variants (DGV), and et al. The pathogenicity of the candidate CNVs was assessed according to the guidelines outlined by the American College of Medical Genetics (ACMG) for interpretation of sequence variants [9].

### Exome sequencing

Trio-exome sequencing was performed on the proband and parents. Target genes were captured by SureSelect Human All Exon V6 (Agilent, San Diego, USA) and enriched. The libraries were quantified and sequenced on Novaseq6000 platform (Illumina, San Diego, USA) with 150 bp pair-end sequencing mode. The clean sequencing reads were aligned to Hg19 reference genome using BWA (https://github.com/lh3/bwa). GATK (https://software.broadinstitute.org/gatk/) were employed for variant calling. Variants annotation and interpretation were conducted by ANNOVAR (https://annovar.openbioinformatics.org/en/latest/). Based on the variant annotations, candidate SNVs/Indels associated with genotypes were identified and confirmed by Sanger sequencing. The variants were classified according to the American College of Medical Genetics and Genomics (ACMG) category.

### Sanger sequencing

Sanger sequencing was performed to confirm the suspected variants identified by Trio-exome sequencing in the family. Primers were designed by Primer5 software. Primer P1 (P1F: TAATGTTTCAGGTCGTGTTCTTT, P1R: GGCAGTTTAAGTATTAACTTGTAGCT) was used to amplify c.2314del (p.M772*). Primer P2 (P2F: TTTATGTGTAACCTTCCTTAGTCCTT, P2R: AACTCACCTTCACAGACTGGCT) was used to amplify c.1415T > G (p.V472G). Target DNA fragments of the family members were amplified by PCR: initial denaturation at 95 °C for 5 min, followed by 34 cycles at 95 °C for 20 s, 60 °C for 20 s and 72 °C for 20 s and a final hold at 72 °C for 4 min. The PCR products were purified and sequenced using ABI 3730xl with the BigDye™ Terminator Cycle Sequencing Kit (Applied Biosystems, Foster, CA, USA). Sanger sequencing results were analyzed by chromas software compared with reference sequence in GenBank (NM_153704.6).

### Protein model

Use AF-Q5HYA8-F1-model_v4 as template [10, 11], models are computed by the SWISS-MODEL server homology modelling pipeline (Waterhouse et al.) which relies on ProMod3, an in-house comparative modelling engine based on OpenStructure [12, 13].

### Diagnostic results

**Fig. 2:**
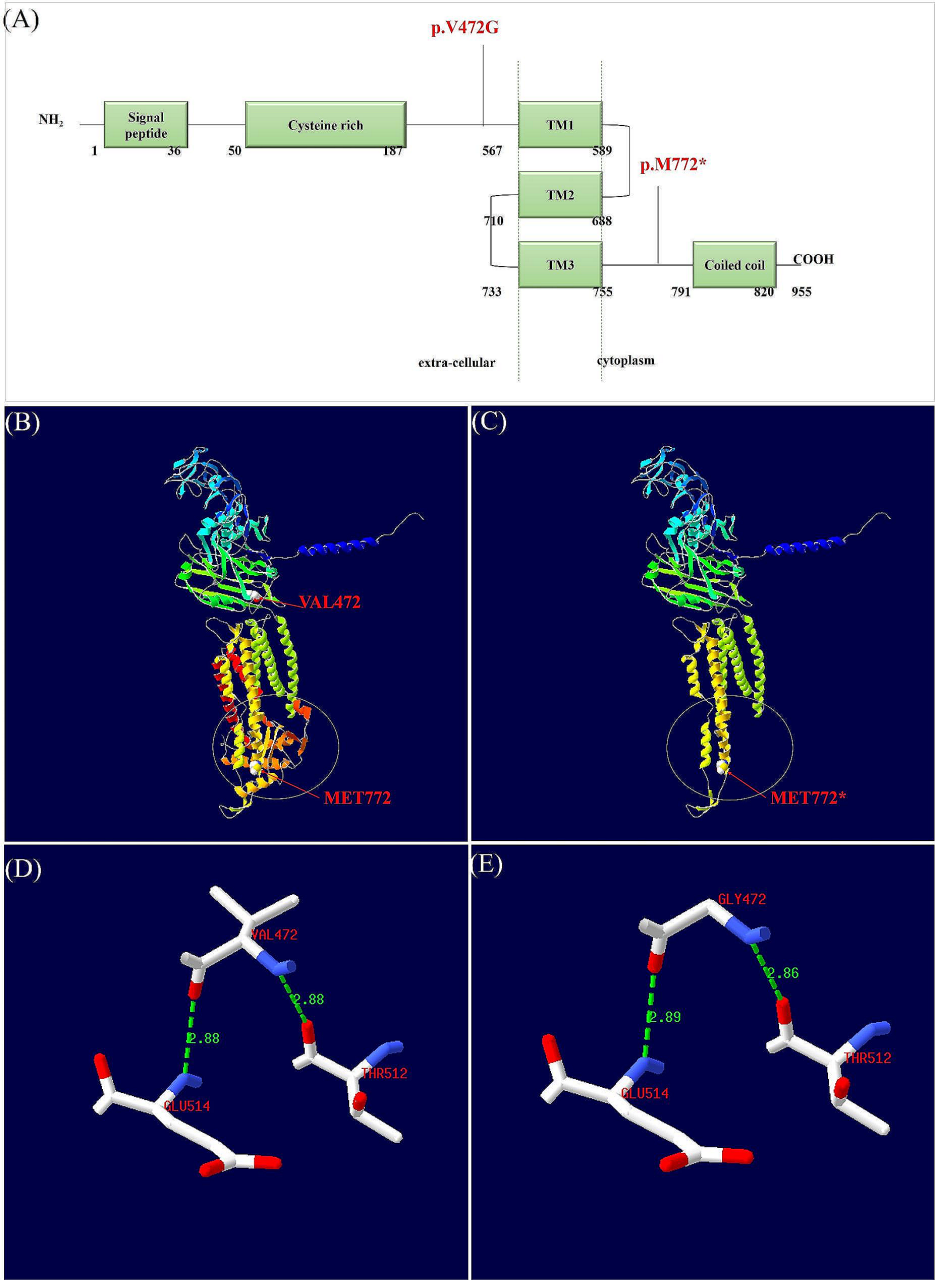
(**A**) The structure of meckelin protein encoded by *TMEM67* gene and the location of the variations. (**B**) Three-dimensional structure diagram of meckelin and the location of the variations. (**C**) Display of protein truncation caused by nonsense mutations M772*. (**D**, **E**) Hydrogen bonds between amino acid residue 472 and surrounding residues 512, 514

No chromosomal abnormality was found in the CNV-seq results (Fig. 1E, F). Trio-exome sequencing revealed a compound heterozygote of *TMEM67* gene in proband: NM_153704.6: c.1415T > G (p.V472G) and c.2314del (p.M772*). c.1415T > G (p.V472G) in exon 14 was paternally inherited, while c.2314del (p.M772*) in exon 22 was maternally inherited. The healthy sister was wild type in the two site. Both of the variants were not reported in HGMD (https://www.hgmd.cf.ac.uk/ac/index.php) and ClinVar (https://www.ncbi.nlm.nih.gov/clinvar/) previously, confirmed by Sanger sequencing (Fig. 1D). The intrauterine phenotype of II5 was consistent with Meckel syndrome caused by the *TMEM67* gene (PP4).

c.2314del (p.M772*) located in the intracellular region of TMEM67 protein, generated a premature stop codon and truncated protein (Fig. 2A, B, C) (PVS1). The frequency of the variant was not found in gnomAD (https://gnomad.broadinstitute.org/), EXAC (https://gnomad.broadinstitute.org/), 1000 G (https://www.internationalgenome.org/) and local database (PM2_Supporting). c.2314del (p.M772*) was classified as “pathogenic” (PP4 + PM2_Supporting + PVS1).

c.1415T > G (p.V472G) located in extra-cellular region was predicted harmful by multiple software (REVEL: 0.8, MetaRNN: 0.8824, MVP: 0.9994, MutationTaster: 0.9999, SIFT: 0.002) (PP3_Moderate). c.1415T > G was classified as “likely pathogenic” (PP4 + PM2_Suppoting + PP3_Moderate + PM3). Unfortunately, genetic testing of the other three adverse pregnancy samples was not possible.

p.M772* leaded to a premature stop codon resulting in loss of “Coiled coil” domain (Fig. 2A, B, C) in cytoplasm. Compared the model carrying p.V472G with AF-Q5HYA8-F1-model_v4, mild changes in hydrogen bonding distance were found between amino acid residue 472 and 512, 514 (Fig. 2D, E). Hydrogen bonding distance between amino acid residue GLY472 and THR512 was short than wild type, while distance between amino acid residue GLY472 and GLU514 was longer than wild type.

## Discussion

*TMEM67* encodes the transmembrane protein meckelin localizing to the primary cilium and to the plasma membrane. *TMEM67* regulates canonical Wnt/β-catenin signaling pathway in the developing cerebellum [14]. Pathogenic mutations in *TMEM67* gene result in dysfunction of primary cilia during early embryogenesis. Primary cilia play an important role in tissue development and signal transduction, with mutations in ciliary-associated genes resulting a broad spectrum of human disease phenotypes [15]. Biallelic variants in the *TMEM67* gene cause COACH syndrome 1 (#216,360), Joubert syndrome 6 (#610,688), Meckel syndrome 3 (#607,361), Nephronophthisis 11 (#613,550), RHYNS syndrome (#602,152) and Bardet-Biedl syndrome 14, modifier of (#615,991). MKS3 causes by *TMEM67* gene is the most severe ciliopathy with almost 100% mortality rate [16], central nervous system abnormalities (occipital encephalocele, hydrocephalus, anencephaly, holoprosencephaly, Dandy–Walker syndrome), bilateral renal polycystic kidneys with cystic dysplasia and polydactyly. Other related conditions are lip/palate cleft, heart defects, genital anomalies, liver fibrosis, and skeletal defects. The presence of earlier-onset oligohydramnios may lead to intrauterine death [17]. MKS3 has a clinical and genetic overlap with other viable ciliopathies, especially Joubert syndrome and Joubert syndrome-related disorders [17, 18].

*TMEM67* gene encodes meckelin protein with an extracellular N-terminus containing a signal peptide and a cysteine rich domain, a transmembrane portion and an intracellular C-terminus including a coiled-coil domain [18]. Genotype-phenotype correlation analysis based on literatures show that biallelic null variants in *TMEM67* gene is more common in lethal MKS3 than milder Joubert syndrome [19]. The distribution of pathogenic missense variants along *TMEM67* gene mainly clustered in the extra-cellular region, such as cysteine rich region, and the transmembrane regions [19].

In our report, c.2314del (p.M772*) generates a premature stop codon that is expected to induce nonsense-mediated mRNA decay (NMD) mechanisms. p.M772* leaded to the loss of “Coiled coil” domain. Multiple variants in “Coiled coil” domain (c.2397T > C, p.Asp799Asp; c.2439G > A, p.Ala813Ala) have been reported relation to MKS [18, 20]. It means a correct “Coiled coil” domain is necessary for meckelin. Multiple null variants (p.K853*, p.E848*, p.N854Kfs*5 and p.L897Ifs*64) in downstream of M722 have been previously reported in MKS cases, indicating that N-terminal loss can also affect normal function of *TMEM67* gene. The truncated protein produced by p.M772* affected the normal gene function. c.1415T > G (p.V472G) locates in extra-cellular region with unknown function. The prediction of protein structure showed that the variant leaded to mild changes in the hydrogen bond distance between amino acid residues. In earlier studies, hydrogen bonds were known to stabilize the structures of proteins and shorter distances imply stronger binding [21, 22]. The changes of the hydrogen bond distance might affect the stability of the meckelin. Pathogenic missense variants from exon 8 to exon 15 of *TMEM67* gene occur simultaneously with null variants at the trans position is common in lethal MKS3 cases [18].

The process of clarifying the genetic cause of RPL in this family is very sad. This family selected for genetic diagnosis, until experienced forth times spontaneous abortions and intrauterine death caused by fetal structural malformations. In this process, the mother suffered great physical and psychological trauma. If prenatal WES testing and corresponding genetic counseling can be carried out early in the family, it would be of great help to prevent recurrence of adverse pregnancies.

In this study, we regrettably were not able to perform genetic testing on II1, II3 and II4. This also exposed the deficiencies in current clinical work. For families with multiple pregnancy failures, adequate genetic counseling and the preservation of unexplained abortion samples were required. In the future, we hope to conduct further research on the mechanism between the *TMEM67* gene and recurrent pregnancy loss.

In conclusion, this report shows the importance of exome sequencing in fetal with RPL, expands *TMEM67* gene variant spectrum related to MKS3. The family in the study experienced multiple adverse pregnancy outcomes. *TMEM67* gene explains the genetic cause of fetus with RPL, provides the opportunity for preimplantation genetic testing and avoids the same adverse pregnancy outcomes in next pregnancy. Furthermore, detailed prenatal phenotypes provide evidence for prenatal WES testing.

## Data Availability

All data generated or analysed during this study are included in this published article.
